# Impact of the Diversity in the 5′ UTR on Translation Regulation

**DOI:** 10.1002/wrna.70060

**Published:** 2026-10-02

**Authors:** Giulia Cardamone, Melanie Flohr, Irina Bode, Tobias Schmid

**Affiliations:** ^1^ Institute of Biochemistry I, Faculty of Medicine Goethe University Frankfurt Frankfurt am Main Germany; ^2^ German Cancer Consortium (DKTK) Partner Site Frankfurt Frankfurt Germany

## Abstract

The 5′ untranslated region (UTR) of mRNAs is crucial to regulate translation initiation. Diversity in the 5′ UTR can be created by alternative transcription start site (TSS) selection and alternative splicing. Alternative TSS directly impact the first exon length or alternative first exon usage, while alternative splicing further contributes to generating different 5′ UTR isoforms by for example, cassette exons inclusion, intron retention, and alternative 5′ or 3′ splice sites. Alternative TSS and alternative splicing in the 5′ UTR directly affect translation efficiency by generating different isoforms that may include or exclude regulatory sequence elements and secondary structures in the 5′ UTR. Among the regulatory elements, 5′ UTRs may harbor translation‐inhibitory upstream open reading frames (uORFs) as well as internal ribosome entry sites (IRESs), which are able to recruit the ribosome in a cap‐independent manner. Both uORF‐ and IRES‐mediated mechanisms contribute to target‐specific protein production, especially under stress conditions associated with an inhibition of global translation. Mechanisms generating different 5′ UTR isoforms therefore represent an additional sophisticated layer to regulate transcript‐selective translation efficiency.

## Graphical abstract

Alternative transcription start site selection and alternative splicing events within the 5′ untranslated region may result in the inclusion or exclusion of translation‐initiation regulatory sequences and structures, thereby providing another layer of transcript‐selective regulation of the translation efficiency, especially in the context of stress responses.

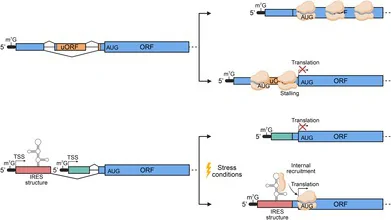

## 1 Introduction

Translation is a tightly controlled process, which is predominantly regulated at the level of initiation (Jackson et al. 2010). Bulk translation of eukaryotic mRNAs occurs in a cap‐dependent manner, where, upon binding to the 5′ 7‐methyl‐guanosine (m^7^G) cap, the eukaryotic initiation factor 4E (eIF4E) recruits additional initiation factors, including the helicase eIF4A and the scaffolding protein eIF4G. When the eIF4F complex is formed, the 43S pre‐initiation complex, containing the small ribosomal subunit and the ternary complex (holding eIF2α and methionine tRNA), is recruited, and the so‐formed 48S initiation complex assumes ribosomal scanning of the 5′ UTR for a start codon (Hinnebusch 2014; Sonenberg and Hinnebusch 2009). In addition to the global regulation of translation, for example, by modulation of the availability of eIF4E via 4E‐binding proteins (4EBPs) (Ruggero et al. 2004), various *cis*‐elements for a transcript‐specific regulation of translation are encoded within the 5′ untranslated region (UTR) of mRNAs (Gebauer et al. 2012; Grayeski et al. 2022; Zeng et al. 2024). The length of human 5′ UTRs is extremely variable, with an estimated median length of 210–218 nucleotides, although some 5′ UTRs may contain thousands of nucleotides (Leppek et al. 2018; Pesole et al. 2001). Interestingly, genes depending on a tight dosage control appear to have relatively long 5′ UTRs (Wieder et al. 2024). The most complete database of 5′ UTRs is represented by UTRdb 2.0, including more than 500 species (Lo Giudice et al. 2023). Of note, about 40% of the human genes contain first exons that are completely non‐coding (i.e., corresponding entirely to the 5′ UTR), with an average length of 151 nucleotides (Davuluri et al. 2001). Consequently, mechanisms contributing to altered first exons or differential splicing can directly affect the diversity of 5′ UTRs. Specifically, alternative transcription start site (TSS) selection, acting at the transcriptional level, and post‐transcriptional alternative splicing, govern the landscape of available 5′ UTRs. While longer 5′ UTRs have been associated with reduced translation efficiency (Wang et al. 2016), alternative TSS and alternative splicing can also impact translation by controlling the presence or absence of regulatory sequences and structural elements, including upstream open reading frames (uORFs) and internal ribosome entry sites (IRESs). These regulatory features are crucial to fine‐tune translation of specific mRNAs under stress conditions, where the cells undergo inhibition of global translation and need a rapid way to adjust protein expression of selected transcripts (Williams and Rousseau 2024).

This review focuses on how mechanisms generating 5′ UTR diversity represent an additional regulatory layer to modulate translation efficiencies of alternative isoforms via inclusion or exclusion of translation‐regulatory elements. We provide a unique focus on transcriptional (alternative TSS) and post‐transcriptional (alternative splicing) mechanisms generating 5′ UTR diversity and their direct consequences on translation efficiency mainly acting on uORFs and secondary structures, including IRESs. While previous reviews focused on how sequence and structural elements regulate translation (Hinnebusch et al. 2016; Leppek et al. 2018) or how genetic variants in the 5′ UTR may disrupt them (Wieder et al. 2025), here we provide insights on the functional consequences of reshaping the 5′ UTR through alternative TSS and alternative splicing mechanisms.

## 2 Mechanisms Generating Diversity in the 5′ UTR

### 2.1 Alternative Transcription Start Site (TSS)

Alternative TSS usage is a widespread mechanism allowing transcription to initiate at different positions within the same gene (Alfonso‐Gonzalez and Hilgers 2024). Transcription initiation is mediated by a complex interaction of transcription factors and the transcription machinery at the core promoter, which ranges 50 nucleotides upstream and downstream of the TSS (Haberle and Stark 2018). Promoter activity is further regulated by the integration of signals from distal regulatory elements and epigenetic modifications, including DNA methylation and histone modification (Heintzman et al. 2007; Weber et al. 2007). More than 50% of human protein‐coding genes bear alternative promoters, which can be classified in sharp promoters, with a defined TSS (usually TATA box–enriched promoters), and broad promoters (usually CpG islands‐rich promoters), with multiple TSSs within the same cluster (Carninci et al. 2006; Kimura et al. 2006). Distant alternative promoters commonly result in 5′ UTRs having alternative first exons, whereas when alternative core promoters are in close proximity, the resulting 5′ UTRs may share the same first exon differing primarily in length; in this case, the 5′ UTRs are defined as tandem (Figure 1A) (Qin et al. 2018; Zhao et al. 2024).

**FIGURE 1:**
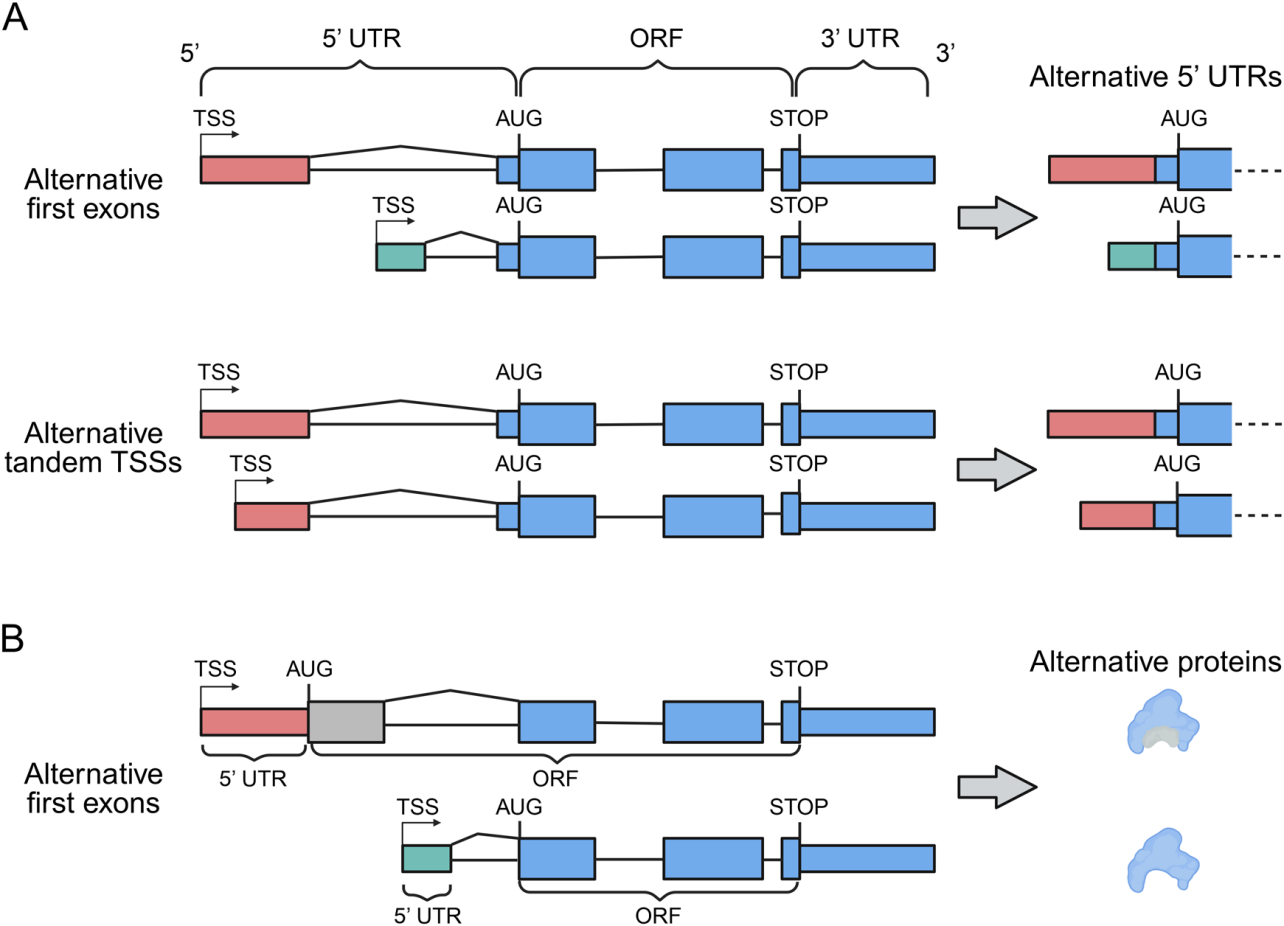
Different types of alternative transcription start site (TSS) selection. (A) Schematic representation of the regulation of alternative first exons and alternative tandem TSSs and their corresponding different 5′ untranslated region (UTR) isoforms. (B) Schematic representation of the impact of alternative first exons including or excluding parts of the coding region, resulting in proteins with different N‐termini. Exons are represented by boxes (open reading frame (ORF)—wide; UTRs—narrow); introns are represented by lines. Constitutive exons are colored in light blue, alternative regions of the 5′ UTR in red and green. The alternative coding region and the corresponding alternative N‐terminus are depicted in gray (B).

Alternative TSSs may also derive from a cluster within the same broad core promoter, resulting in 5′ UTRs that differ in length by only few nucleotides (Kawaji et al. 2006). Besides generating different 5′ UTR isoforms, alternative TSS usage may also directly affect the proteome by generating proteins with different N‐termini, if different isoforms include different coding regions (Figure 1B) (Rosenstiel et al. 2007; Zaika et al. 2002). Among the molecular mechanisms underlying alternative TSS selection, regulation mediated by transcription factors promoting initiation at different sites and alteration of the chromatin state have been shown to play a major role (de Klerk and Hoen 2015). With respect to epigenetic mechanisms, it was shown that DNA methylation of certain CpG sites around first exon regions was associated with tandem TSS usage, leading to the usage of distal TSSs, that is, allowing for transcription to start more 5′ relative to the proximal TSS (Zhao et al. 2024). Global epigenetic changes, induced upon inhibition of DNA methyltransferases and histone deacetylases, were also shown to affect tandem alternative TSS usage, increasing distal TSS usage of several genes (Zhao et al. 2024). Interestingly, DNA methyltransferase and histone deacetylase inhibitors also appeared to induce transcription from alternative, non‐annotated TSSs (Brocks et al. 2017). Along the same lines, hypoxia was recently shown to induce TSS‐switching through complex epigenetic mechanisms, leading to changes in the nucleosome occupancy and positioning around TSSs (Watt et al. 2025). The fusion oncogene *RUNX family transcription factor 1* (*RUNX1*)/*RUNX1 partner transcriptional co‐repressor 1* (*RUNX1T1*) provides an example where epigenetic regulation affects alternative TSS: its binding to specific genomic sites, known to change chromatin accessibility, represses the usage of alternative TSSs, whereas its knockdown increases the chromatin accessibility of alternative TSSs, leading to their transcriptional activation (Grinev et al. 2021). Along these lines, an upstream TSS of the *nuclear receptor subfamily group 2 group F member 2* (*NR2F2*) gene was shown to be inactivated by DNA methylation. Demethylation occurring during metastatic melanoma progression leads to the usage of the upstream TSS and to the expression of an alternative protein isoform with a different N‐terminus associated with melanoma cell survival and metastasis (Davalos et al. 2023). TSSs might also derive from transposable elements, functioning as alternative promoters for nearby host genes, which has been demonstrated to contribute to tissue‐specific gene expression (Zhang et al. 2026).

To map actively used TSSs experimentally, Cap Analysis of Gene Expression (CAGE) and variants thereof are the most commonly used methods (Carninci et al. 2006). These methods are based on capturing and subsequent sequence analysis of the capped 5′ ends of RNAs (Takahashi et al. 2012). The largest CAGE‐based TSS atlas in human tissues, promoted by the functional annotation of the mammalian genome (Fantom) 5 project, revealed a widespread and cell‐specific use of alternative TSSs and provided evidence for an average of four TSSs per gene (Forrest et al. 2014). Additional methods to capture the 5′ UTR end of transcript include RAMPAGE (Batut and Gingeras 2013), oligo‐capping (Suzuki and Sugano 2003), and GRO‐cap (Core et al. 2014); for a benchmarking analysis see (Adiconis et al. 2018).

Functionally, both alternative first exons and tandem TSS usage were demonstrated to represent widespread mechanisms in cancer. Alternative promoters, whose activity significantly differs between cancer and normal samples, were shown to produce alternative first exons across multiple cancer types, for example, in the case of the tumor suppressor genes *testin LIM domain protein* (*TES*) or *speckle‐type BTB/POZ protein* (*SPOP*) (Demircioğlu et al. 2019). With respect to tandem TSS usage, similar patterns of exon usage were found in cancers with similar histological features, for example, *signal transducer and activator of transcription 6* (*STAT6*) displays higher levels of distal TSS usage both in colon and rectum adenocarcinoma compared to other cancer types (Zhao et al. 2024).

### 2.2 Alternative Splicing

In contrast to alternative TSS selection, alternative splicing acts on the post‐transcriptional level allowing for the diversification of the transcriptome through the expression of different mRNAs from a single gene (Black 2003). Deep RNA‐sequencing analyses estimated that more than 95% of human multi‐exon genes undergo alternative splicing (Merkin et al. 2012; Pan et al. 2008). Alternative splicing is rather well characterized for the coding regions, where it contributes to the generation of distinct protein isoforms. Alternative splicing within the UTRs, on the other hand, rather affects mRNA stability, translation, and localization (Kajdasz et al. 2022; Mockenhaupt and Makeyev 2015). Interestingly, as many as 28% of human 5′ UTRs contain at least one intron (Pesole et al. 2001) and alternative splicing occurs in up to 22% of human 5′ UTRs (Modrek et al. 2001). In fact, several types of alternative splicing events have been described for various 5′ UTRs (Figure 2) (Mockenhaupt and Makeyev 2015).

**FIGURE 2:**
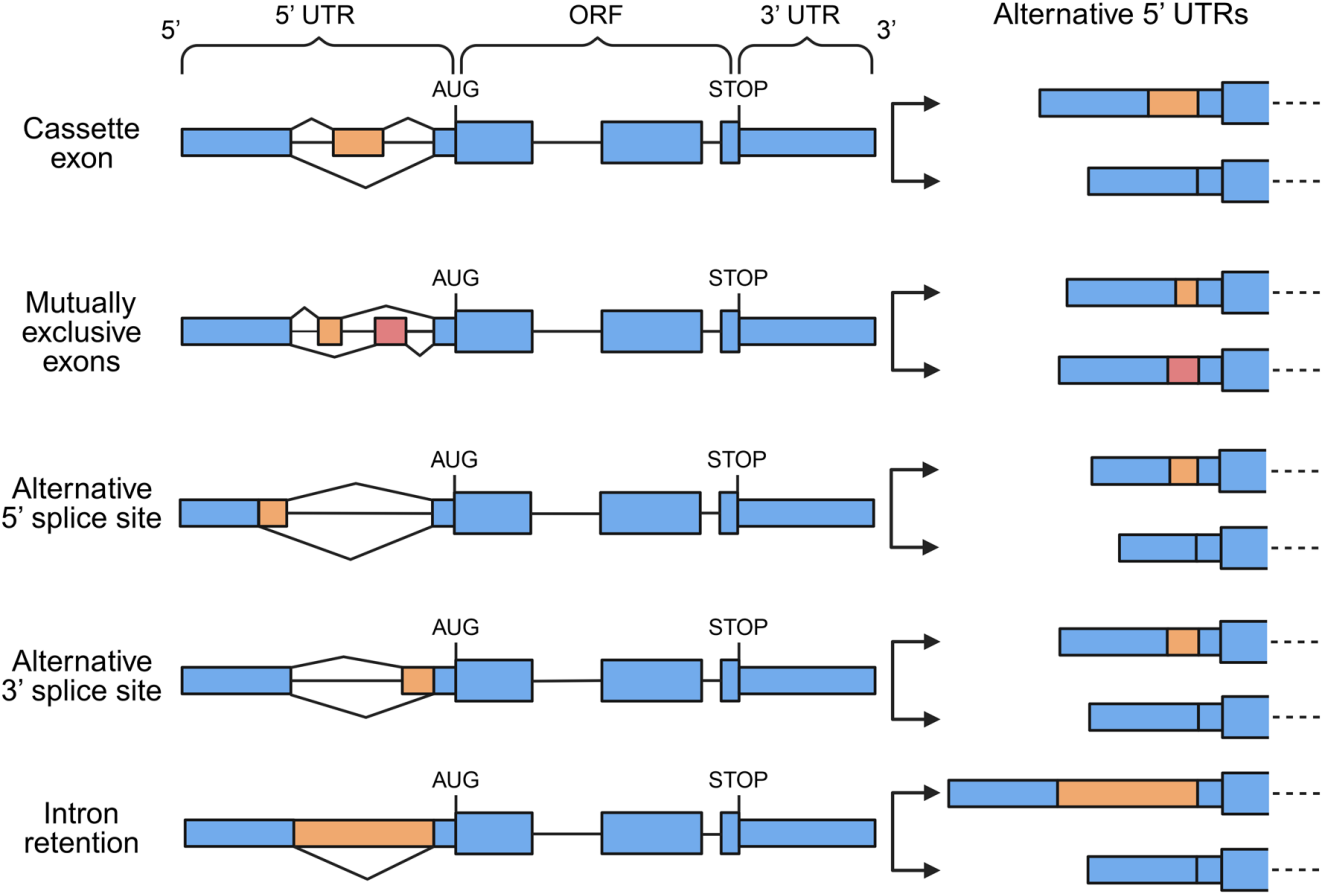
Different types of alternative splicing events in the 5′ untranslated region (UTR). Schematic representation of the different types of alternative splicing events (cassette exon, mutually exclusive exons, alternative 5′ and 3′ splice sites, and intron retention) and their corresponding 5′ UTR isoforms. Exons are represented by boxes (open reading frame (ORF)—wide; UTRs—narrow); introns are represented by lines. Constitutive exons are colored in light blue, alternative exons in orange and red.

Cassette exons, which are either included or skipped in the transcript, are the most frequent alternative splicing modality in human transcripts (Wang et al. 2008). Mutually exclusive exons represent an alternative splicing event where only one exon among a cluster of candidate exons is included in the mature mRNA (Hatje et al. 2017). In the 5′ UTR, this event would require the presence of additional up‐ and downstream non‐coding exons, making it a rather rare event due to the limited length of 5′ UTRs. Alternative 5′ and 3′ splice sites refer to the possibility that alternative donor splice and acceptor splice sites are used during alternative splicing, respectively, thus generating isoforms having either up‐ or downstream exons of different lengths (Koren et al. 2007). Finally, intron retention refers to the inclusion of a complete and unspliced intron in the mature mRNA (Grabski et al. 2021).

Alternative splicing regulation is mediated by a complex interaction of *cis*‐acting sequence elements and *trans*‐acting factors. In detail, splicing enhancers, that is, regulatory exonic or intronic sequence elements, can promote the recruitment of the spliceosome and consequently exon inclusion, whereas splicing silencers interrupt the assembly of the splicing machinery, thereby favoring exon skipping. These elements are recognized by *trans*‐acting factors, including serine and arginine‐rich splicing factors (SRSFs) as well as heterogeneous nuclear ribonucleoproteins (hnRNPs) (Marasco and Kornblihtt 2023). Splicing enhancers are commonly bound by SR proteins, which favor exon inclusion, whereas hnRNPs bind to splicing silencers and are generally considered to be splicing repressors (Busch and Hertel 2012). Of note, several alternative splicing‐associated RNA‐binding proteins also play a role in other post‐transcriptional processes. For example, SRSF1 was shown to modulate both translation and alternative splicing of the same targets (Maslon et al. 2014). Similarly, hnRNP A1 not only controls alternative splicing processes, but also regulates RNA stability and translation (Feng et al. 2022), and the translation regulator programmed cell death 4 (PDCD4) was recently shown to also alter splicing of various transcripts (Dezi et al. 2026). The multifaceted role of these RNA‐binding proteins further strengthens the concept of the functional interconnection between alternative splicing and translation.

Methods to obtain a transcriptome‐wide map of alternative splicing events were first based on short‐read RNA‐sequencing and transcript reconstruction analyses (Feng et al. 2011). The development of long‐read RNA‐sequencing methods nowadays allows for direct assessment of sequences of entire transcript isoforms (Ament et al. 2025). Several alternative splicing analysis tools are available, both for short‐ and long‐read analyses; a comparative analysis of the available alternative splicing analysis tools, with their strengths and limitations, is reviewed in (Tran et al. 2025). Long‐read sequencing methods provided novel, unprecedented insights into the close interconnection between alternative TSS and mRNA processing steps, including alternative splicing and alternative polyadenylation (Alfonso‐Gonzalez et al. 2023; Anvar et al. 2018). Noteworthy, for a high percentage of genes a coupling of alternative TSS with alternative splicing events was identified, also involving exons not in close proximity to the TSS (Anvar et al. 2018). Thus, when alternative TSS is combined with alternative splicing events within the 5′ UTR, this further expands the landscape of 5′ UTR isoforms.

The co‐transcriptional nature of alternative splicing has been debated, with evidence suggesting that the removal of introns may occur during transcription (Kornblihtt et al. 2013). Among the proposed mechanisms, recruitment of splicing factors to transcription sites and the RNA polymerase II kinetics play a prominent role (Boumpas et al. 2023; Kornblihtt et al. 2013). Indeed, it has been demonstrated that some splicing factors can be recruited by the transcription machinery, as in the case of SRSF3 and hnRNP L (de la Mata and Kornblihtt 2006; Huang et al. 2012). With respect to the RNA polymerase II kinetics, it has been proposed that slower rates of RNA polymerase II elongation are associated with exon inclusion, whereas accelerated elongation rates lead to exon skipping, as shown for *fibronectin 1* (*FN1*) and *neural cell adhesion molecule 1* (*NCAM1*) cassette exons (Kadener et al. 2001; Schor et al. 2009). Recently, it has been shown that splicing may also influence transcription initiation through mechanisms including the local recruitment of the transcription machinery by splicing factors. This in turn influences promoter selection and RNA polymerase II elongation, underscoring the close interconnection between these processes (Fiszbein et al. 2019; Tellier et al. 2020). Therefore, methods combining long‐read sequencing with 5′ cap‐trapping are crucial to obtain a refined overview of the landscape of 5′ UTR alternative splicing isoforms with the exact TSS (Weber et al. 2023).

Since both alternative TSS and alternative splicing have also been shown to affect translation efficiency (Niederer et al. 2022; Sugimoto and Ratcliffe 2022; Wang et al. 2016; Weber et al. 2023), methods were developed combining polysome profiling either with RNA‐seq analyses to study the impact of alternative splicing isoforms on translation (Floor and Doudna 2016; Sterne‐Weiler et al. 2013) or with 5′ end RNA sequencing to investigate the impact of alternative TSS on translation and map the exact TSS of each isoform (Sugimoto and Ratcliffe 2022; Wang et al. 2016). However, a unique method able to assess the impact of both alternative splicing and alternative TSS on translation at the same time remains to be developed.

## 3 Translation Regulation

Sequence or structural elements within the 5′ UTR are crucial for transcript‐specific regulation of translation initiation. Specifically, under conditions that suppress global translation, such as cellular stress, translation of selected mRNAs can be maintained by uORF‐ or IRES‐dependent mechanisms (Holcik and Sonenberg 2005; Jackson et al. 2010).

### 3.1 Upstream Open Reading Frames (uORFs)

uORFs are short ORFs defined by a start codon in the 5′ transcript leader sequence with an in‐frame stop codon. Global analyses revealed that active translation of uORFs can reduce production of the associated main ORF‐encoded proteins by up to 80% (Calvo et al. 2009). Noteworthy, ribosome profiling, still the gold standard to determine translation efficiency on a transcriptome‐wide level (Ingolia et al. 2009), intrinsically also detects uORFs due to their ribosome occupancy (Johnstone et al. 2016) and in the meantime both bioinformatics tools (Scholz et al. 2019) as well as databases of uORFs are available (Manske et al. 2023). uORFs are extremely common in eukaryotic transcriptomes, indicating that they likely constitute an important layer of post‐transcriptional gene regulation (Whiffin et al. 2020). Along these lines, uORFs appear to be especially enriched in essential genes, such as oncogenes and other key regulatory factors (Ye et al. 2015). Indeed, one of the main functions of uORFs appears to be the coordination of translation of stress response genes (Starck et al. 2016), where they mostly repress translation of the main ORF under basal conditions (Palam et al. 2011). Stress then leads to a switch of translation from uORF to main ORF, thereby allowing for rapid regulation of stress‐response genes (Chen et al. 2025). Importantly, the relative translation of uORFs and their associated main ORFs is strongly influenced by both the strength of the start codon (i.e., AUG > > CUG > GUG) (Kearse and Wilusz 2017) and the local sequence context of either start codon, that is, the Kozak sequence (Kozak 1989).

As depicted in Figure 3, uORFs can reduce translation of the associated main ORFs by numerous mechanisms: (A) If a uORF overlaps (out‐of‐frame) with the associated main ORF, reduced translation of the latter is due to the fact that the scanning ribosome never even reaches the start codon of the main ORF (Mihailovich et al. 2007; Rogers et al. 2004). This has been well‐characterized in the case of the *cyclin‐dependent kinase inhibitor 1A* (*CDKN1A*) transcript, for example (Kim et al. 2012), but genome‐wide analyses suggest numerous additional transcripts in which uORFs overlap with the main ORF (Calvo et al. 2009; McGillivray et al. 2018). Importantly, if an upstream, alternative start codon is in‐frame with the main ORF, it does not represent a classical uORF and rather constitutes a 5′ extension of the main ORF, leading to the production of an N‐terminally extended protein (Fritsch et al. 2012; Morrison and Rissland 2025). (B) If the uORF is not overlapping with the main ORF, translating ribosomes may stall or dissociate at the stop codon of the uORF, thus, scanning ribosomes again do not reach the main ORF (Lin et al. 2019). Such a mode of regulation was recently suggested in the case of *tumor necrosis factor alpha‐induced protein 2* (*TNFAIP2*), where inefficient TNFAIP2 protein synthesis was observed even though *TNFAIP2* mRNA showed substantial ribosome‐association. Indeed, the majority of *TNFAIP2* mRNA appeared to be bound by two ribosomes within a uORF in the translation‐inhibited state. Considering the length of the uORF (36 nucleotides), this was indicative of ribosome stalling at the stop codon of the uORF, yielding colliding ribosomes within the uORF (Scholz et al. 2020). Noteworthy, uORF‐dependent regulation of TNFAIP2 appeared to be especially important in the context of myeloid differentiation and, thus, constitutes one of only few examples that are not centered on the cancer context. Interestingly, ribosome stalling in uORFs is also central to the concept that uORF‐encoded peptides might interact with the ribosome or with the nascent peptide itself to induce translational pausing in response to specific metabolites or stress signals (Mize et al. 1998). Mechanistically, such stalling might occur if the uORF‐encoded peptide interacts with the ribosomal exit tunnel, thereby altering ribosome conformation and impairing ribosome progression along the mRNA (Shanmuganathan et al. 2019). In line, recent analyses have identified several so‐called conserved peptide uORFs that repress main ORF translation in a sequence‐dependent manner (Dever et al. 2020; Takahashi et al. 2020). Yet, since the peptide sequences of different uORFs within one organism are generally not highly conserved, a translation‐regulatory function of uORF‐derived peptides likely only accounts for a small fraction of the uORF‐mediated effects. Owing to their small size, uORF‐derived peptides are rather challenging to detect, and, thus, elucidation of their exact functions remains challenging (Lee et al. 2021). (C) Even if the 40S ribosomal subunit remains attached to the mRNA after translation termination at the uORF stop codon, and starts to scan for another start codon (Skabkin et al. 2013), initiation factors (including the ternary complex) have to be recruited anew to allow for effective reinitiation at a downstream start codon (Bohlen et al. 2020; Mohammad et al. 2017). As these factors are already used during initiation at the uORF start codon, some essential components of the initiation machinery might be depleted, consequently reducing reinitiation efficacy (Russell et al. 2023). Such a mode of regulation has been shown for example in *growth arrest and DNA damage‐inducible protein 34* (*GADD34*) and *protein phosphatase 1 regulatory subunit 15B* (*PPP1R15B*) (Young et al. 2015). Notably, reinitiation is a highly transcript‐specific mechanism, and some mRNAs, such as *inhibitor of bruton tyrosine kinase α* (*IBTKα*) and oncoprotein *mouse double minute 2* (*MDM2*), have evolved uORF arrangements that specifically favor reinitiation at their main ORF under defined conditions (Andreev et al. 2024; Baird et al. 2014).

**FIGURE 3:**
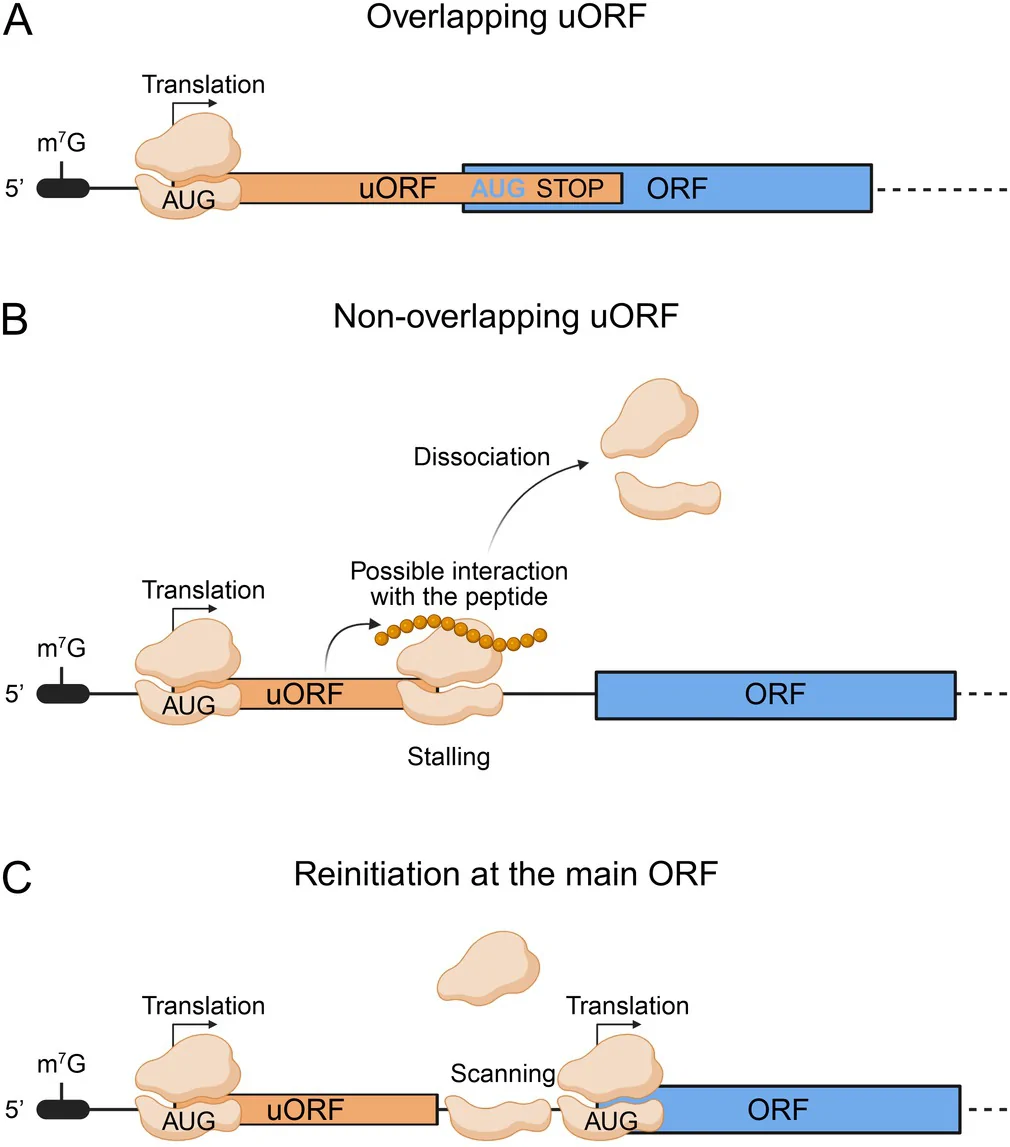
Translational inhibition by upstream open reading frames (uORFs). (A) Translation of uORFs overlapping with the main ORF prevents translation of the main ORF, as scanning ribosomes never reach the start codon of the latter. (B) Translation of uORFs residing entirely in the 5′ untranslated region (UTR) inhibits translation of the main ORF by ribosome dissociation or stalling upon translation termination at the uORF stop codon. uORF‐encoded peptides can further induce ribosome stalling at the uORF to inhibit translation of the main ORF. (C) If ribosomes remain associated with the mRNA after uORF termination, they can reinitiate translation at the downstream main ORF, if required initiation factors are recruited in due time.

Importantly, the above‐described mechanisms of uORF‐mediated translational regulation of the main ORF are not mutually exclusive, that is, a single transcript with multiple uORFs may combine several of the above‐mentioned strategies to fine‐tune translation of the main ORF.

Since uORFs are generally considered to repress translation of the associated main ORFs, timely inactivation of the inhibitory uORFs is essential to allow for rapid synthesis of uORF‐repressed proteins. Thus, not surprisingly, uORF‐mediated regulation of protein synthesis is of specific relevance in stress responses (Moro et al. 2021), and most mechanisms of uORF regulation have been studied extensively in the context of the integrated stress response (ISR), including ER stress (Andreev et al. 2015; Harding et al. 2000; Starck et al. 2016). A common feature of these stimuli is that they activate so‐called ISR sensor kinases, which phosphorylate eIF2α and consequently reduce the availability of the ternary complex (Sudhakar et al. 2000).

*Activating transcription factor 4* (*ATF4*) is one of the most extensively studied examples of uORF‐mediated translational control and nicely illustrates the versatility of this regulatory concept. In detail, *ATF4* mRNA contains two critical uORFs: Under non‐stress conditions, ribosomes efficiently translate the short first uORF and then reinitiate at the inhibitory second uORF, which overlaps out‐of‐frame with the main ORF, thereby preventing ATF4 protein synthesis (Smirnova et al. 2024; Vattem and Wek 2004). During cellular stress, phosphorylation of eIF2α reduces ternary complex availability, which reduces reinitiation competence. This allows ribosomes to bypass the second uORF and instead reinitiate at the main ORF start codon, consequently increasing *ATF4* translation despite a suppression of global protein synthesis (Lu et al. 2004; Vattem and Wek 2004). Consequently, *ATF4* translation is rapidly and strongly upregulated under stress conditions and contributes to the orchestration of cellular adaptations to stress (May and McManus 2022; Young and Wek 2016). Other stress‐responsive mRNAs, such as *ATF5* and *DNA damage inducible transcript 3* (*DDIT3*, *CHOP*), use similar uORF‐based strategies to achieve preferential translation when eIF2α is phosphorylated (Palam et al. 2011; Watatani et al. 2008; Zhou et al. 2008).

Thus, uORFs constitute an important sequence‐encoded *cis*‐regulatory element allowing for transcript‐specific regulation of translation initiation.

### 3.2 Internal Ribosome Entry Sites (IRESs)

IRESs are functional RNA elements allowing for translation initiation in a cap‐independent manner by ribosome recruitment to other parts of the 5′ UTR or directly to the start codon. They were first discovered in picornaviruses, including poliovirus and encephalomyocarditis virus (Jang et al. 1988; Pelletier and Sonenberg 1988), and characterized to be complex secondary RNA structures within the 5′ UTR of RNAs. Since viral RNAs are uncapped, they rely exclusively on internal translation initiation by direct ribosome recruitment without the requirement of cellular initiation factors to bypass the host cell defense and to ensure translation of viral RNAs by the host cell machinery (Lee et al. 2017). In contrast, translation initiation of eukaryotic mRNAs is predominantly facilitated by recruitment of the initiation machinery to the 5′ m^7^G cap of the mRNAs. Since all eukaryotic mRNAs can be translated in a cap‐dependent manner, an mRNA‐specific regulation of translation initiation is commonly achieved by cap‐independent modes. Moreover, while cap‐mediated translation is often inhibited by cellular stress conditions, such as hypoxia and nutrient limitation (Holcik and Sonenberg 2005), IRES‐mediated translation ensures continued expression of proteins relevant for survival or adaptative processes (Holcik et al. 2000). Hundreds of experimentally validated IRESs are reported in IRESbase and IRESite databases (Mokrejš et al. 2006; Zhao et al. 2020). While viral IRESs are well characterized as having common properties such as long and highly structured 5′ UTRs with conserved motifs among virus families, cellular IRESs remained rather poorly understood due to technological constraints (Stoneley and Willis 2004). One of the reasons being that bicistronic vectors, a fundamental tool for IRES research, harbor the risk of detecting cryptic splicing or cryptic promoter activities after insertion of the IRES sequence into the vector (Baranick et al. 2008; Jackson 2013). Nevertheless, the establishment of rigorous control experiments, such as RT‐qPCR of the reporter mRNAs or inclusion of promoterless vectors, but also the use of circular RNA vectors allowed for ruling out cryptic splice and promoter activities and, thus, unambiguous identification of eukaryotic IRESs (Chu et al. 2021; van den Akker et al. 2021). Nevertheless, cellular IRESs received more attention in recent years, owing to the realization that eukaryotic IRESs are enriched in mRNAs encoding for proteins involved in critical cellular processes including cell survival, stress response, and mitosis, and, thus, are associated with various diseases (Sriram et al. 2018). For example, IRESs have been identified in oncogenic mRNAs, such as *c‐MYC*, *B‐cell lymphoma 2* (*BCL‐2*), or anti‐apoptotic *X‐linked inhibitor of apoptosis* (*XIAP*), ensuring continued translation of these mRNAs under otherwise translation‐inhibitory conditions, and thereby promoting tumor progression (Holcik et al. 1999; Nanbru et al. 1997; Sherrill et al. 2004). So far, 774 eukaryotic IRESs have been reported (Zhao et al. 2020). The first eukaryotic IRES was identified in the mRNA encoding immunoglobulin heavy‐chain binding protein *heat shock protein family A* (*Hsp70*) *member 5* (*HSPA5*, *BiP*), which is a stress‐induced chaperone (Gething 1999; Macejak and Sarnow 1991). Increased BiP levels due to upregulated IRES activity were described during viral infections (Buchkovich et al. 2010; Najarro et al. 2024).

The activity of cellular IRESs appears to depend both on RNA structures and interacting RNA‐binding proteins, so‐called IRES *trans*‐acting factors (ITAFs) (Figure 4). In fact, most cellular IRESs are considered to contain rather complex secondary RNA structures, such as stem‐loops, hairpins, RNA G‐quadruplexes, or pseudoknots, which influence translation in a highly context‐dependent manner (Leppek et al. 2018). For example, RNA G‐quadruplexes are stable, four‐stranded structures that are formed in guanine‐rich sequences within the 5′ UTR of mRNAs (Millevoi et al. 2012). They are often associated with translational repression by sterically preventing the recruitment of the 43S pre‐initiation complex or by blocking ribosome scanning (Beaudoin and Perreault 2010). However, a G‐quadruplex was reported within the *vascular endothelial growth factor* (*VEGFA*) IRES to be related to an increased IRES activity by direct recruitment of the 40S ribosomal subunit (Bhattacharyya et al. 2015; Niu et al. 2022). Pseudoknots represent another example of regulatory RNA structures. They are complex secondary RNA structures characterized by at least two intercalated stem‐loops (Pleij 1990). While pseudoknots play an important role in various viral IRESs, including hepatitis C virus (Wang et al. 1995), so far a pseudoknot was only identified as part of a larger structure in the cellular *c‐MYC* IRES. Of note, despite the detailed structural characterization of the overlapping pseudoknot within the *c‐MYC* IRES, its regulatory function for the IRES activity still remains unclear (Le Quesne et al. 2001).

**FIGURE 4:**
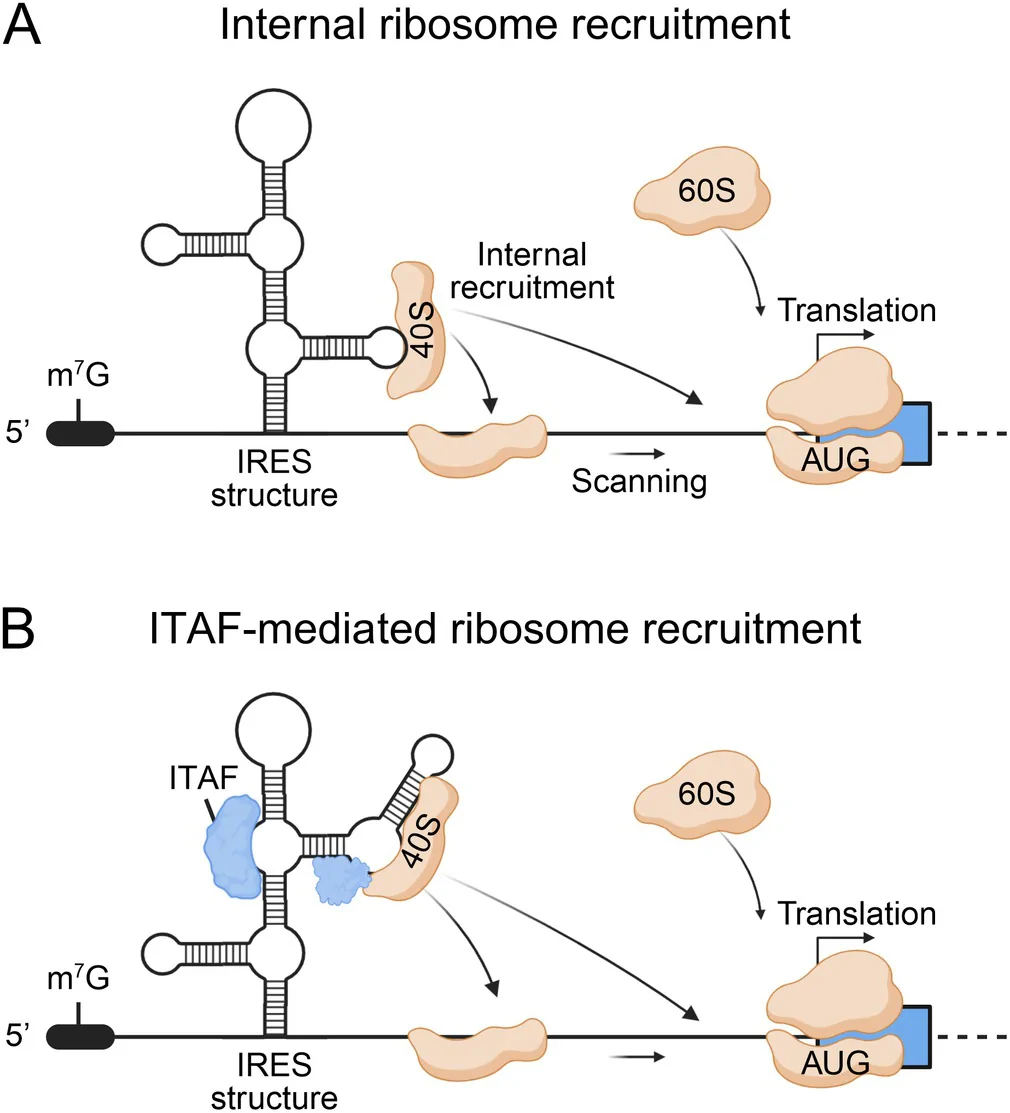
Internal ribosome entry site (IRES)‐dependent translation initiation. (A) IRES‐dependent translation initiation is an alternative mode independent of cap recognition. IRESs are considered to be complex secondary RNA structures within the 5′ UTR, which can directly recruit the ribosomal 40S subunit. Subsequently, the 40S subunit either recruits initiation factors to facilitate scanning for a start codon or, alternatively, the 40S subunit can be directly placed on the start codon. After start codon recognition, the 60S ribosomal subunit is recruited to allow for translation initiation, elongation, and termination. (B) IRES‐mediated translation initiation commonly depends on supporting proteins, the so‐called IRES *trans*‐acting factors (ITAFs). Binding of ITAFs to the IRES can lead to conformational changes of the mRNA structure, thereby facilitating (or preventing) ribosome recruitment.

IRES‐constituting structures are commonly bound by ITAFs, which might allow for direct, internal recruitment of ribosomes (King et al. 2010). The interaction with specific ITAFs can also contribute to structural rearrangements of IRES structures; thus, either activating or inactivating the associated IRES (Figure 4B). Indeed, ITAFs are involved in most cases of IRES‐mediated translation as they can stabilize secondary RNA structures or induce structural changes to facilitate ribosome recruitment (Lewis and Holcik 2008). Examples include the IRES of the proto‐oncogene *c‐MYC*, which is dysregulated in various types of cancer (Shi et al. 2022). A point mutation within the *c‐MYC* IRES of multiple myeloma samples was indeed reported to be associated with increased *c‐MYC* expression (Chappell et al. 2000). This mutation was suggested to induce a stem‐loop structure within the *c‐MYC* IRES, which enhances binding of ITAFs. The IRES of the *apoptotic protease‐activating factor 1* (*APAF1*) is another example of the important role of ITAFs to initiate IRES‐activating structural changes. Here, binding of *cold shock domain‐containing E1* (*CSDE1*, *UNR*) leads to structural rearrangements allowing for binding of polypyrimidine tract‐binding protein 1 (PTBP1), which subsequently induces structural changes eventually allowing for ribosome recruitment (Mitchell et al. 2003). In line with the essential function of ITAFs for IRES‐mediated translation and the important role of IRESs for the translation of numerous tumor‐associated proteins, tumorigenic conditions were shown to commonly lead to dysregulation of various ITAFs. For example, while the ITAFs RNA helicase A (RHA) and translational control protein 80 (TCP80) control the IRES‐mediated translation of the tumor‐suppressor *tumor protein 53* (*TP53*), their expression is low in tumor cells; thus, restricting p53 expression and consequently appropriate DNA damage responses (Halaby et al. 2015). In contrast, increased levels of the MDM2 proto‐oncogene, which has a moonlighting function as an ITAF, enhance IRES‐mediated translation of the oncogene *XIAP*, leading to the resistance of cancer cells to irradiation (Gu et al. 2009).

While the majority of cellular IRESs remain rather poorly characterized, the recent advent of new technologies opened new opportunities for detailed analyses of cellular IRESs. Importantly, RNA structure probing techniques such as parallel analysis of RNA structure (PARS), selective 2′‐hydroxyl acylation analyzed by primer extension and mutational profiling (SHAPE‐MaP), or dimethyl sulfate mutational profiling with sequencing (DMS‐MaPseq) in combination with visualization tools including RNAfold or VARNA have been optimized to allow for high‐throughput, but also targeted in‐depth characterization of RNA structures in vivo (Darty et al. 2009; Kertesz et al. 2010; Lorenz et al. 2011; Siegfried et al. 2014; Zubradt et al. 2017). The possibility to determine structures in vivo at great depth allows for the assessment of stimulus‐ and/or ITAF‐dependent changes in relevant structures. Moreover, novel vectors have been developed to allow for direct comparisons of cap‐ and IRES‐dependent translation from the same mRNA transcript (van den Akker et al. 2021), and even have been adapted to real‐time imaging of the activities on a single‐molecule level (Koch et al. 2020). Such novel vectors are expected to shed further light on the exact contribution of IRES‐dependent translation to the entire translational output of individual transcripts and might finally overcome existing reservations regarding the existence of cellular IRESs, which are mostly due to their physiologically reasonable, rather low activities in comparison to viral IRESs (Bert et al. 2006; Gilbert 2010). And lastly, high‐throughput approaches were developed to comprehensively screen for the presence of IRESs across large sets of cellular 5′ UTRs (Weingarten‐Gabbay et al. 2016). Nevertheless, since the exact constituents of most eukaryotic IRESs, that is, the contributing mRNA structure and/or sequence motifs, still remain largely elusive, future studies should focus on generating unambiguous proof for the relevant *cis*‐regulatory elements of individual IRESs. Detailed knowledge of these will then also allow for the integration of *trans*‐acting proteins, and eventually allow for therapeutic exploitation of this highly transcript‐specific mode of translational regulation.

Importantly, structure‐associated (IRESs) and sequence‐determined (uORFs) translation‐regulatory features within the 5′ UTRs are not necessarily independent of each other and, in fact, can cooperate to control translation. For example, the *cationic amino acid transporter 1* (*CAT1*) 5′ UTR contains an IRES and a uORF embedded within this IRES. Under amino acid starvation, translation of the uORF causes a rearrangement of the 5′ UTR structure, which activates the IRES and consequently enhances cap‐independent translation of CAT1 (Yaman et al. 2003). Thus, it appears critical to understand how the diversity of 5′ UTRs impinges on the translational output.

## 4 Functional Consequence of Alternative TSS and Alternative Splicing on Translation

Several studies suggest that different 5′ UTR isoforms generated by both alternative TSS and alternative splicing comprise different translation efficiencies. Yet, while some 5′ UTR isoforms of specific transcripts were associated with an altered translation efficiency in pathophysiological contexts, the underlying molecular mechanisms remain to be characterized (Chang et al. 2019; Daian et al. 2020; Ghilardi et al. 1998; Ishii et al. 2013; Kramer et al. 2013; Lundell et al. 2007; Manek et al. 2020; McDade et al. 2007; Roberts et al. 1997; Shalev et al. 2002; Sharma et al. 2023). In those cases where different translation efficiencies appeared to be associated with changes in alternative TSS or alternative splicing, it commonly involved the inclusion or exclusion of regulatory sequences or structural elements. Indeed, the presence of uORFs and/or 5′ cap‐adjacent stable secondary RNA structures was associated with decreased translation efficiency by several studies (Jagannatha et al. 2024; Sugimoto and Ratcliffe 2022; Wang et al. 2016).

### 4.1 uORF‐Mediated Mechanisms

Interestingly, more uORFs were found in the alternative regions of human 5′ UTRs, resulting from alternative TSS or alternative splicing, compared to constitutive regions (Resch et al. 2009). In line, a high proportion of transcripts with longer 5′ UTRs compared to the annotated ones was observed during human CD4 T‐cell activation. Among these, a longer 5′ UTR isoform of the *C‐X‐C motif chemokine receptor 5* (*CXCR5*) gene was identified, deriving from an alternative promoter, showing a reduced translation efficiency. Indeed, this novel variant contained three predicted uORFs, upon mutation of which, CXCR5 protein expression increased. Thus, the decreased translation efficiency of the alternative isoform was attributed to the presence of translation‐inhibitory uORFs in the longer isoform (Woolley et al. 2025). Similarly, Zhao and colleagues demonstrated widespread dysregulation of tandem TSS usage in tumors, which was associated with altered mRNA stabilities and translation efficiencies. They further identified two tandem TSS isoforms of the *translocase of inner mitochondrial membrane 13* (*TIMM13*) transcript in lung cancer cells, which differed in the length of their 5′ UTRs. The shorter isoform, produced by a proximal TSS, was associated with increased translational output compared to the longer isoform, produced by a distal TSS. Reduced translation efficiency of the longer isoform was attributable to two putative uORFs (Zhao et al. 2024). Alternative TSS‐dependent alternative 5′ UTR isoforms of *Nanog Homeobox (NANOG*), *Snail Family Transcriptional Repressor 1 (SNAI1*), and *Nodal Growth Differentiation Factor (NODAL*) transcripts were also shown to account for increased translation of the respective mRNAs in breast cancer cells in response to hypoxia. In the case of *NODAL*, a putative uORF was identified in one of the alternative longer isoforms, and experimentally validated to contribute to reduced translation under hypoxia by mutation analyses (Jewer et al. 2020). In line with the tight association of uORF‐dependent regulation with stress, two 5′ UTR isoforms of *ATF5* resulting from alternative first exon usage were found to be translation‐inhibitory, but behaved differently in response to stress conditions. In fact, translational repression of only one isoform was overcome by stress conditions, such as amino acid limitation, and mutation of the respective uORF included in this isoform was shown to abolish this effect (Watatani et al. 2008).

In addition to alternative TSS, differential 5′ UTR isoforms exclusively derived from alternative splicing were also shown to impact translation due to the inclusion of uORFs (Figure 5A). For example, the retention of an intron within the *STN1 subunit of CST complex* (*STN1*) 5′ UTR was shown to result in the inclusion of three uORFs, for which the translation‐restricting capacities were validated again by mutational analyses in a reporter system. Reduced translation efficiency due to AS‐dependent inclusion of these inhibitory uORFs was observed in lung adenocarcinoma and associated with increased survival (Huang et al. 2023). Similarly, de Breyne and colleagues demonstrated that upon B‐cell activation, alternative splicing leads to the inclusion of an alternative exon in the 5′ UTR of the *RBMX like 1* (*RBMXL1*) transcript, which resulted in the creation of a uORF and, consequently, to reduced translation efficiency (de Breyne et al. 2025). Additionally, the inclusion of an alternative exon in the 5′ UTR of *adiponectin receptor 1* (*ADIPOR1*) was associated with reduced translation efficiency due to the presence of inhibitory uORFs (Ashwal et al. 2011).

**FIGURE 5:**
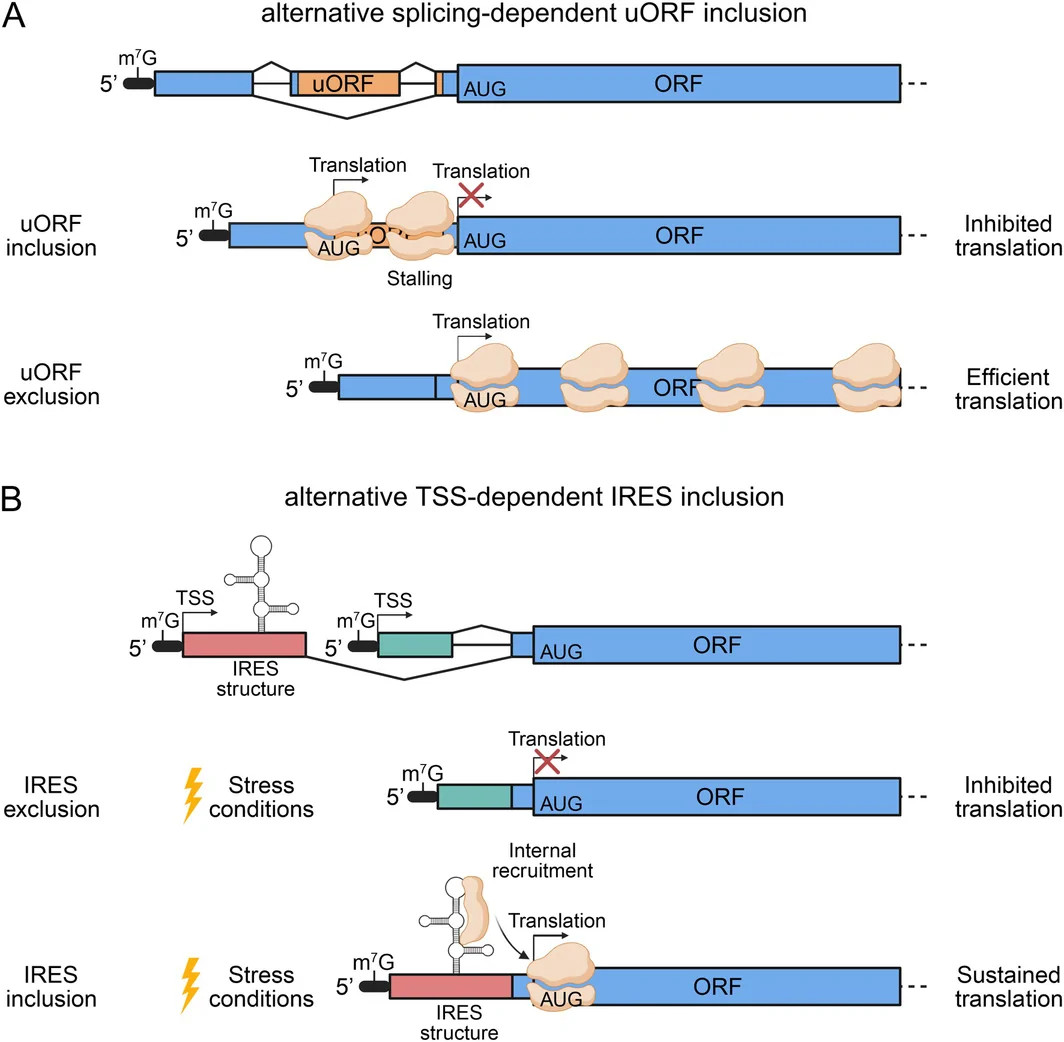
Impact of alternative splicing or alternative transcription start site (TSS) selection on the presence of translation regulatory elements. (A) Schematic representation of the inclusion and exclusion of an alternative exon containing an upstream open reading frame (uORF). In the depicted example, the inclusion of a uORF results in ribosome stalling and the consequent inhibition of translation, whereas the isoform not including the alternative exon is efficiently translated. (B) Schematic representation of the inclusion or exclusion of an internal ribosome entry site (IRES) element due to the alternative first exon usage mechanism of alternative TSS selection. In the depicted example, under stress conditions, translation is inhibited, whereas the inclusion of the IRES allows for sustained translation. Exons are represented by boxes (open reading frame (ORF)—wide; 5′ untranslated regions (UTR)—narrow); introns are represented by lines. The 5′ m^7^G cap structure is also depicted. Constitutive exons are colored in light blue, alternative exons in orange (A) and red and green (B).

Interestingly, a combination of alternative TSS and alternative splicing generated different 5′ UTR isoforms of the *tribbles pseudokinase 3* (*TRIB3*) transcript, displaying differential translation efficiencies. Again, one of the less efficiently translated isoforms was shown to include an inhibitory uORF, whose mutation increased the translation efficiency of the isoform (Örd et al. 2009).

### 4.2 Secondary RNA Structure‐Mediated Mechanisms

Besides uORFs, 5′ UTR isoforms deriving from alternative TSS and/or alternative splicing were also demonstrated to commonly include or exclude RNA structure elements, including IRESs (Figure 5B).

The *fibroblast growth factor 1* (*FGF1*) transcript displays four 5′ UTR isoforms, resulting from four alternative tissue‐specific promoters, referred to as transcripts A‐D, two of which were shown to bear an IRES (i.e., transcripts “A” and “C” contain IRESs “A” and “C”, respectively) (Martineau et al. 2004). A follow‐up study revealed that during myoblast differentiation cap‐dependent translation is inhibited, but *FGF1* translation is sustained through the activation of IRES “A” (Conte et al. 2009). In contrast, a combination of alternative TSS (an alternative first exon) and alternative splicing (alternative 3′ acceptor site) were shown to be responsible for the removal of the well‐characterized IRES of *XIAP*. While the IRES‐depleted isoform was expressed under normal growth conditions, its translation was inhibited under stress conditions. Under these conditions, cap‐independent translation mediated by the IRES‐bearing longer isoform was enhanced (Riley et al. 2010). Similarly, the transcript of the transcription factor regulator of hematopoiesis *RUNX1* was shown to contain two 5′ UTRs produced by two alternative promoters. The longer isoform was demonstrated to contain a functional IRES element facilitating cap‐independent translation in a bicistronic vector system. While the IRES‐bearing transcript appeared to be translated less efficiently in K562 chronic myeloid leukemia cells under basal conditions, the IRES activity increased substantially during megakaryocytic differentiation (Pozner et al. 2000). Noteworthy, earlier studies already showed that cap‐independent translation of other transcripts, such as *platelet‐derived growth factor subunit b* (*PDGFB*), is enhanced during megakaryocyte differentiation as well (Bernstein et al. 1995). Importantly, the case of *RUNX1* underscores the fine line between common translation‐inhibitory effects of complex RNA structures and their potential role as selective, cap‐independent translation initiation facilitators as in IRESs. Along the same lines, Al‐Zeer and colleagues identified an IRES element with a G‐quadruplex structure in the longer of two 5′ UTR isoforms of the *actin‐related protein 2/3 complex subunit 2* (*ARPC2*) transcript due to alternative first exon usage (Al‐Zeer et al. 2019). Mutation of the G‐quadruplex structure in a bicistronic vector assay reduced translation efficiency, supporting the notion that G‐quadruplexes, besides inhibiting cap‐dependent translation, can support IRES‐mediated, cap‐independent translation (Morris et al. 2010). In line with the translation‐inhibitory effects of G‐quadruplexes, a longer isoform of *ArfGAP with GTPase domain, ankyrin repeat and PH domain 2* (*AGAP2*), resulting from alternative TSS within the same cluster at the core promoter, was shown to include a G‐quadruplex contributing to the reduced translation efficiency in chronic myeloid leukemia and prostate cancer cells (Surani et al. 2022).

Importantly, the impact of eukaryotic IRESs likely remains underestimated as their characterization is technically challenging. Specifically, while the identification of underlying RNA structures and relevant RNA‐binding protein interactions alone requires rather complex technical approaches, a combination of both cooperative layers of regulation awaits the development of novel technologies.

### 4.3 Combination of uORF‐ and Secondary RNA Structure‐Mediated Mechanisms

In addition to the separate occurrence of 5′ UTR sequence‐ or structure‐driven changes in translational regulation, a combination of both mechanisms was described to explain the different translation efficiencies of specific isoforms. For example, in the *membrane spanning 4‐domains A1* (*MS4A1*) transcript, encoding the CD20 protein, an important target for immunotherapy, alternative splicing events in the 5′ UTR were shown to produce some inefficiently translated isoforms due to the presence of uORFs and an RNA stem‐loop structure repressing translation. Importantly, the balance between translation‐deficient and ‐competent isoforms appeared to modulate CD20 protein levels and the subsequent responses to CD20‐directed immunotherapies (Ang et al. 2023). In the case of *glycyl‐tRNA synthetase 1* (*GARS1*), Alexandrova and colleagues characterized two 5′ UTR isoforms derived from alternative TSS usage, where the longer 5′ UTR, besides including a uORF, was shown to confer IRES activity. The presence of the uORF in the longer isoform was suggested to prevent the use of a main ORF start codon located within the uORF, that is, the start codon of an overlapping main ORF, which is specific to the mitochondrial form of the enzyme, whereas it allows for reinitiation at a start codon specific to the cytosolic one, although the mechanism linking the IRES activity to this regulation has not been further elucidated (Alexandrova et al. 2015). For *discs large MAGUK scaffold protein 1* (*DLG1*), alternative splicing events in the 5′ UTR resulted in a longer isoform associated with reduced translation efficiency due to the inclusion of a uORF. RNA structure prediction analysis further revealed the presence of a stable stem‐loop in the longer isoforms, suggesting that the combination of the uORF and stable secondary RNA structures both contribute to the reduced translation efficiency (Cavatorta et al. 2011). Similarly, alternative splicing in the 5′ UTR was shown to produce two 5′ UTR isoforms in the *ETS transcription factor ELK1* (*ELK1*) transcript, both containing a uORF and a stable stem‐loop with no IRES activity, yet the longer isoform appeared to be more translationally repressive than the shorter one (Araud et al. 2007). Follow‐up studies showed that the longer isoform in fact displayed a more pronounced attenuation of the uORF‐mediated reinitiation frequency (Rahim et al. 2012). A rather complex case is provided by *serpin family A member 1* (*SERPINA1*), where a combination of alternative TSS and alternative splicing events in the 5′ UTR produces a high number of 5′ UTR isoforms, including or excluding various uORFs and secondary RNA structures. Corley and colleagues demonstrated that secondary RNA structures, predicted by SHAPE‐MaP analyses, changed the accessibility of individual uORFs to their respective Kozak sequence, modulating their translation efficiency and their overall effect on translation (Corley et al. 2017). This example highlights how a complex interplay between uORFs and secondary structures might be involved in translation regulation and could be fine‐tuned by the inclusion and exclusion of elements through alternative TSS and alternative splicing.

## 5 Conclusion

Taken together, regulation of the diversity of 5′ UTRs of transcripts by alternative TSS selection or alternative splicing provides an important layer to control the availability of translation initiation‐regulatory sequence and/or structural features, thereby contributing to the target‐specific protein production especially under conditions of global translational inhibition. Since stress‐associated conditions are commonly found in disease contexts, it appears of major importance that the exact impact of alternative TSS and alternative splicing on translational deregulation, especially of disease‐relevant proteins, is elucidated in detail to allow for rational design of potential intervention strategies. Of note, therapeutic strategies targeting 5′ UTR elements, for example, with antisense oligonucleotides and small molecules (Balaratnam et al. 2023; Liang et al. 2017), are currently being investigated. It should be further noted that while the majority of studies published so far focus on cancer, the characterization of the regulation and impact of the 5′ UTR landscape on translation regulation in other physiological or pathophysiological stress conditions is vastly understudied. Thus, future studies should be directed and expanded towards other contexts, such as development, immune responses, and aging.

## Author Contributions

**Giulia Cardamone:** conceptualization, writing – original draft, writing – review and editing, funding acquisition. **Melanie Flohr:** conceptualization, writing – original draft, writing – review and editing. **Irina Bode:** conceptualization, writing – original draft, writing – review and editing. **Tobias Schmid:** conceptualization, funding acquisition, writing – original draft, writing – review and editing, supervision.

## Funding

This work was supported by the Deutsche Forschungsgemeinschaft (GRK 2336 TP6, SCHM2663/7) (TS) and by Goethe University FFF Nachwuchsforscher‐Förderung (GC).

## Conflicts of Interest

The authors declare no conflicts of interest.

## Data Availability

Data sharing not applicable to this article as no datasets were generated or analysed during the current study.
